# Evaluation of internally cooled radiofrequency ablation targeting multiple shoulder nerves for chronic shoulder pain relief and function restoration: a prospective study

**DOI:** 10.1093/pm/pnaf016

**Published:** 2025-02-27

**Authors:** Mitsukuni Yoshida, Peter K Park, Loc V Thang

**Affiliations:** Division of Pain Medicine, Department of Anesthesiology, Washington University School of Medicine, St Louis, MO, United States; Division of Pain Medicine, Department of Anesthesiology, Washington University School of Medicine, St Louis, MO, United States; Division of Physical Medicine & Rehabilitation, Department of Orthopaedic Surgery, Washington University School of Medicine, St Louis, MO, United States; Division of Pain Medicine, Department of Anesthesiology, Washington University School of Medicine, St Louis, MO, United States

**Keywords:** shoulder pain, radiofrequency ablation, functional outcomes

## Abstract

**Objective:**

Shoulder pain is a prevalent musculoskeletal disorder, affecting up to 70% of adults over their lifetimes. Recently, radiofrequency ablation (RFA) has gained attention as a treatment for joint pain. This study aims to evaluate the efficacy of internally cooled RFA (icRFA) in alleviating shoulder pain and improving both qualitative and quantitative measures of shoulder function.

**Design:**

Prospective cohort study.

**Subjects:**

Patients with chronic shoulder pain (*n* = 35) who responded positively to diagnostic blocks of the suprascapular nerve (SSN), axillary nerve (AN), and lateral pectoral nerve (LPN).

**Methods:**

Patients underwent icRFA targeting the SSN, AN, and LPN. Treatment response was assessed with the numeric rating scale (NRS) for pain, the American Shoulder and Elbow Surgeons (ASES) score, and active range of motion (AROM) in flexion and abduction. Outcomes were measured at baseline and at 1, 3, and 6 months after icRFA. Patient characteristics were compared between responders and nonresponders to icRFA treatment, and correlation analyses were conducted to explore the relationship between pain relief and functional improvement.

**Results:**

NRS pain levels significantly decreased at 1 month (Adjusted [Adj.] *P* *<* .0001), 3 months (Adj. *P* *<* .0001), and 6  months (Adj. *P* = .0002) after icRFA. ASES score improved at 1 month (Adj. *P* *<* .0001), 3 months (Adj. *P* *<* .0001), and 6 months (Adj. *P* *<* .0001) after icRFA. Flexion AROM improved at 1 month (Adj. *P* *<* .0001), 3 months (Adj. *P* *<* .0001), and 6 months (Adj. *P* = .0139) after icRFA. Abduction AROM improved at 1 month (Adj. *P* *<* .0001), 3 months (Adj. *P* *<* .0001), and 6 months (Adj. *P* *<* .0001) after icRFA.

**Conclusion:**

iccRFA targeting the SSN, AN, and LPN is a safe and effective intervention, providing significant improvements in pain, functional activities of daily living, and AROM of the shoulder for at least 6 months.

## Introduction

Shoulder pain is a prevalent condition in the general population, accounting for approximately 15 in 1000 primary care visits and affecting up to 70% of individuals over their lifetimes.^1^ It is one of the most common musculoskeletal disorders in adults, with etiologies often rooted in acromioclavicular joint osteoarthritis, glenohumeral osteoarthritis, and rotator cuff pathology.^2^ These conditions frequently lead to pain exacerbated by activity and motion, resulting in significant impairments in activities of daily living.^3–5^ Consequently, shoulder pain not only diminishes the quality of life and functional capacity but also imposes a substantial economic burden, with osteoarthritis-related expenditure in the United States estimated at $72 billion in 2008–2011.^6^^,^^7^

The management of shoulder pain is typically multimodal, involving physical therapy, pharmacotherapy, intra-articular steroid injection, or surgery.^8^ Intra-articular steroid injections remain the most common interventional treatment despite inconclusive evidence for their long-term efficacy and well-documented adverse effects of steroids, including hyperglycemia, weight gain, hypertension, fluid retention, osteoporosis, and immunosuppression.^9^ Although steroid injections are generally considered effective, their pain relief duration is highly variable and patient dependent. Furthermore, although shoulder arthroplasty can offer functional improvement, persistent pain after the surgery is not uncommon, and patient eligibility for the surgery is often limited by comorbid conditions.^10–12^ Given these limitations, there is a pressing need for alternative interventional treatments for this widespread issue.

Radiofrequency ablation (RFA) has shown promise in managing pain syndromes associated with other joints such as the spine and knees, with growing evidence supporting its use.^13–15^ Cadaveric studies have identified the main nerves innervating the glenohumeral joint—namely, the suprascapular nerve (SSN), the axillary nerve (AN), and the lateral pectoral nerve (LPN)—as potential RFA targets.^16^ Further research by Eckmann et al. and Tran et al. has delineated the articular divisions of the 3 nerves, including motor and sensory components, and established safe anatomic zones for RFA to avoid compromising the motor function while treating pain.^16–18^ A 2019 systemic review by Orhurhu et al. aggregated data from 18 studies on RFA for shoulder pain and concluded that RFA could benefit patients with this condition.^19^ However, most of the included studies were case reports or case series, and nearly all focused on pulse RFA (pRFA) targeting the SSN alone. A more recent systematic review and meta-analysis by Pushparaj et al. in 2021 found no significant difference between conventional medical management and pRFA of the shoulder. Similar to the work of Orhurhu et al., that review primarily analyzed studies focusing on pRFA of the SSN.^20^ In 2020, Eckmann et al. provided data supporting the utility of icRFA through case series that included both traditional RFA and icRFA of the SSN, AN, and LPN.^21^ Furthermore, Stogicza et al. explored the use of ultrasound-guided cryoablation targeting the acromial, superior, and inferior branches of SSN, the nerve to the subscapularis, and the LPN.^22^ Most recently, Santi et al. retrospectively analyzed and demonstrated the effectiveness of icRFA targeting SSN, AN, and LPN in reducing pain levels, disability, and pain medication use.^23^ These findings underscore the need for further investigation into the optimal RFA techniques for shoulder pain, including the identification of specific nerve targets that maximize pain reduction and functional outcomes in both the short and long terms.

The present clinical study aimed to assess the efficacy of icRFA targeting the SSN, AN, and LPN in patients with various shoulder pathologies, contributing to the growing body of evidence supporting icRFA as a viable treatment option for chronic shoulder pain. We evaluated the degree and duration of pain relief, assessed improvement in function (with the American Shoulder and Elbow Surgeons Questionnaire), and quantified the active range of motion (AROM). Our research intended to provide qualitative and quantitative evidence supporting icRFA’s utility in improving patient outcome and quality of life.

## Methods

### Study design

This is a prospective, observational study conducted at a single tertiary academic center. A total of 36 shoulder RFA encounters were enrolled, with the first data collection on August 29, 2022, and the last on April 8, 2024. The study was approved by the institutional review board of Washington University in St. Louis School of Medicine (#202207082). The study was conducted according to the tenets of the Declaration of Helsinki. Study participants were recruited through the Washington University Pain Center on a voluntary basis. Written informed consent was obtained from all participants before the study procedure.

### Eligibility criteria

Adult patients over the age of 18 years with any existing shoulder pathology, as evidenced by clinical or radiological diagnosis, were included. Conditions included acromioclavicular osteoarthritis, glenohumeral osteoarthritis, rotator cuff pathology, labral pathology, and adhesive capsulitis. An average daily pain score of greater than 5 on the numeric rating scale (NRS) for the prior 3 months was used as a cutoff for symptom severity, as this level of pain is reported to be severe enough to affect function.^24^ Participants had attempted prior conservative management, including physical therapy, topical or analgesic medications, or corticosteroid injections. All participants demonstrated greater than 50% relief in shoulder pain after diagnostic blocks of the SSN, AN, and LPN. Exclusion criteria included age younger than 18 years, allergy to local anesthetic agents, pregnancy or breastfeeding, active local or systemic infection, active use of anticoagulants, or a history of metastatic cancer.

### Data acquisition and variables

Descriptive statistics, including demographics, pain characteristics, diagnosed conditions, psychiatric comorbidities, and opioid usage, were collected for all patients who completed the study (Table 1). Pain intensities and functional limitations of the shoulder among the study participants were quantified by NRS for pain and the American Shoulder and Elbow Surgeons Questionnaire (ASES).^25–28^ The AROM of shoulder flexion and abduction was measured with a goniometer. Completing the ASES is simple for the patient, as it usually takes less than 5 minutes.^29^ The ASES, as a composite score of pain and function at 1-, 3-, and 6-month follow-up, was the primary outcome in the study, whereas NRS of pain and AROM of shoulder flexion and abduction were secondary outcome measures. All outcomes were measured at baseline before patients had undergone icRFA and then at 1, 3, and 6 months after the procedure.

**Table 1.** Baseline characteristics of the study participants.

Characteristics		
Age, years, range (mean)	53–84	(70.3)
BMI, range (mean)	17.54–54.43	(31.73)
Gender, *n* (%)		
Female	24	(66.7%)
Male	12	(33.3%)
Average NRS pain level, range (mean)	6–10	(7.97)
Duration of pain, months, *n* (%)		
6–9 months	2	(5.9%)
>12 months	34	(94.4%)
Race		
African American	8	(22.2%)
White	28	(77.8%)
Smoking status	8	(22.2%)
Nonsmoker	25	(69.4%)
Current smoker	7	(19.4%)
Former smoker	7	(19.4%)
Psychiatric condition		
None	26	(72.2%)
Anxiety disorders (GAD, PTSD, etc.)	5	(13.9%)
Mood disorders (MDD, bipolar, etc.)	7	(19.4%)
Psychotic disorders (schizophrenia, schizoaffective, etc.)	0	(0%)
Others	0	(0%)

*Abbreviations:* GAD = General Anxiety Disorder; PTSD = Post-traumatic stress disorder; MDD = Major depressive disorder.

### Diagnostic block and icRFA approach

Both diagnostic blocks and icRFA procedures were performed under the supervision of the same attending physician (L.V.T.) to improve the consistency of procedural techniques.

### Diagnostic block

Patients were positioned in the lateral decubitus position, with the affected shoulder facing up. The shoulder area was prepped with chlorhexidine solution anteriorly and posteriorly.

For the SSN and AN, the C-arm was oblique to the ipsilateral side of the posterior shoulder with a caudal tilt to identify the spinoglenoid notch. At the needle entry point, the skin and subcutaneous tissues were infiltrated with 1% lidocaine. The 22-gauge spinal needle was advanced to the posterior osseous rim of the glenoid fossa, lateral and superior to the spinoglenoid notch. With the needle in the correct position, 0.5 mL of 0.5% bupivacaine was injected to block the SSN. With the C-arm in the same position, the patient’s arm was internally rotated until the palm was facing posteriorly to enable identification of the axillary fossa. At the needle entry point, the skin and subcutaneous tissues were infiltrated with 1% lidocaine. The 22-gauge spinal needle was advanced to the most inferior and lateral border of the greater tubercle. With the needle in the correct position, 0.5 mL of 0.5% bupivacaine was injected to block the AN.

For the LPN, the C-arm was oblique to the ipsilateral side of the anterior shoulder with cephalic tilt until the coracoid process was elongated and appeared as a “thumbs-down.” At the needle entry point, the skin and subcutaneous tissues were infiltrated with 1% lidocaine. The 22-gauge spinal needle was advanced to the mid-coracoid process. With the needle in the correct position, 0.5 mL of 0.5% bupivacaine was injected to block the lateral pectoralis. Patients were discharged with an 8-hour pain diary to track their NRS for shoulder pain.

### icRFA

**Figure 1.**
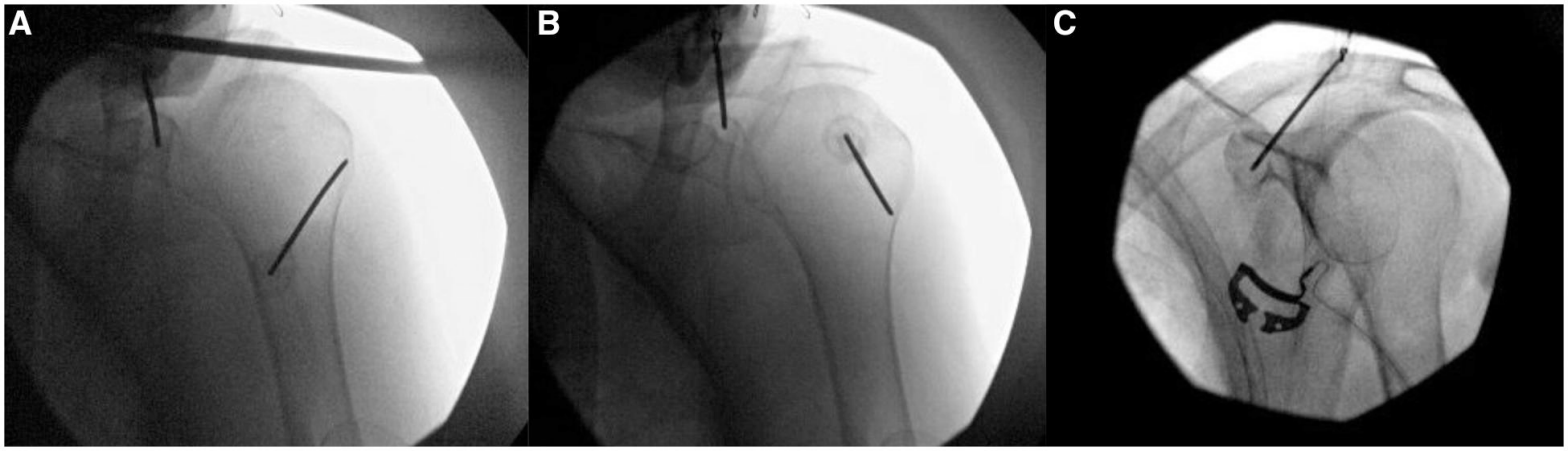
Fluoroscopic images showing the respective target points around the shoulder. Internally cooled radiofrequency ablation (icRFA) technique targeting **(A and B)** the suprascapular nerve (SSN) and axillary nerve (AN) posteriorly and **(C)** the lateral pectoral nerve (LPN) anteriorly. After the first ablation of the SSN and AN in **(A)**, needles targeting the SSN at the glenoid rim were redirected cephalically, and needles targeting the AN at the base of the greater tuber were redirected caudally, as shown in **(B).**

icRFA was performed under fluoroscopic guidance to visualize the target site in a way that was identical to the approach used for the diagnostic blocks. After the skin and subcutaneous tissues were infiltrated with 1% lidocaine at the needle entry point, a 17-gauge 2-mm radiofrequency needle was advanced to the posterior osseous rim of the glenoid fossa, lateral and superior to the spinoglenoid notch. Similarly, a 17-gauge 2-mm radiofrequency needle was advanced to the most inferior and lateral border of the greater tubercle (Figure 1A). Sensory testing at 50 Hz and motor testing at 2 Hz were conducted and confirmed negative at each of the aforementioned points. After negative aspiration, 1 mL of a mixture of 5 mL 4% lidocaine and 1 mL of 4 mg dexamethasone was injected at each location. RFA was then performed at 60 degrees Celsius at the needle tip (which corresponds to 80 degrees Celsius at the tissue level) for 2.5 minutes. The needles were then repositioned slightly more cephalically, and RFA was performed again at this new location at 60 degrees Celsius at the needle tip (which corresponds with 80 degrees Celsius at the tissue level) for 2.5 minutes (Figure 1B).

For the LPN, a 17-gauge 2-mm radiofrequency needle was advanced to the mid-coracoid process (Figure 1C). With the needle in the correct position, sensory testing was conducted at 50 Hz, and motor testing was performed at 2 Hz, and the results were confirmed negative. After negative aspiration, 1 mL of a mixture of 5 mL 4% lidocaine and 1 mL of 4 mg dexamethasone was injected. RFA was then performed at 60 degrees Celsius at the needle tip (which corresponds with 80 degrees Celsius at the tissue level) for 2.5 minutes.

### Statistical analysis and sample size estimation

Data analyses were conducted in R studio (version R-4.2.1) and GraphPad Prism 10 (GraphPad Software). Descriptive statistics were used to summarize the data as appropriate. Normal distribution was assessed from the skewness and kurtosis of the data and verified with the Shapiro-Wilk test. One-way ANOVA was used to analyze the effect of icRFA on the NRS, ASES score, and the degrees of AROM. A mixed model for repeated measures (MMRM analysis) with the Geisser–Greenhouse correction was used to account for missing data and to allow for potential correlation in each outcome measure between time periods. Dunnett’s multiple comparison was used to compare the post-RFA data against pre-RFA data. Unpaired *t*-test and Fischer’s exact test were used to compare the continuous and categorical variables between responders and nonresponders to icRFA, respectively. Responders were defined as participants who received at least 50% maximum pain reduction, according to the NRS, at any time after the icRFA procedure. Spearman correlation analysis was performed to characterize the relationship between pain reduction and functional improvement. All statistical analyses were performed according to the predefined statistical analysis plan.

A power analysis for repeated-measures ANOVA was performed in G*Power v3.1.9.4 to determine the minimum sample size needed to examine the effect of icRFA treatment on pain and motor function of patients’ shoulders, as measured by the change in ASES scores across 4 observations: at baseline and at 1, 2 and 3 months. On the basis of this design and assuming a medium treatment effect (η^2^*P* = .05), an alpha of 0.05, a moderate correlation of 0.30, and a power level of 0.80, a sample of 32 patients is needed to reject the null hypothesis of no treatment effect. To allow for 10–20% attrition due to possible dropouts and missing data, 36 patients were ultimately enrolled. One patient was lost to follow-up, and 35 patients completed the study.

## Results

### Baseline characteristics

Between August 29, 2022, and April 8, 2024, a total of 36 patients with a history of chronic shoulder pain were screened, underwent diagnostic blocks, and were enrolled in the study. One patient was lost to follow-up after the treatment with icRFA. The study was continued with 35 patients, whose baseline characteristics and treatment history are summarized in Tables 1 and 2. The mean age of participants was 70.4 years, ranging from 53 to 84 years, with female predominance (66.7%). Baseline assessments recorded average NRS pain level as 7.97, ranging from 6 to 10. The majority of patients had a history of chronic shoulder pain lasting more than 12 months (94%). The shoulder pathologies of the participants included glenohumeral osteoarthritis (92%), acromioclavicular osteoarthritis (75%), rotator cuff pathology (86%), labral pathology (44%), adhesive capsulitis (42%), biceps tendinopathy (25%), and bursitis pathology (33%) (Table 2). Most patients had undergone non-opioid pharmacotherapy (97.2%) and intra-articular steroid injection (97.2%). The patient demographics in our study represented a typical patient population with shoulder pain who undergo various treatments in our clinic and others.

**Table 2.** Shoulder pain characteristics and treatment history.

Characteristics		
Laterality, *n* (%)		
Left	25	(69.4%)
Right	10	(27.8%)
Both	1	(2.78%)
Localization of pain, *n* (%)		
Generalized	6	(16.7%)
Anterior	3	(8.3%)
Posterior	27	(75%)
Pain radiation, *n* (%)		
No radiation	19	(52.8%)
Radiation	17	(47.2%)
Shoulder pathology, *n* (%)		
Glenohumeral osteoarthritis	33	(91.7%)
Acromioclavicular osteoarthritis	27	(75%)
Rotator cuff pathology	31	(86.1%)
Labral pathology	16	(44.4%)
Adhesive capsulitis	15	(41.7%)
Biceps tendinopathy	9	(25%)
Bursitis pathology	12	(33.3%)
Nonspecific shoulder pain	1	(2.78%)
Treatment history, *n* (%)		
Non-opioid oral medications	35	(97.2%)
Steroid injections	35	(97.2%)
Topical medications	21	(58.3%)
Rotator cuff repair surgery	3	(8.33%)
Massage therapy	11	(30.6%)
Acupuncture	10	(27.8%)
Other (TENS, ultrasound, etc.)	8	(22.2%)
Current opioid use, MME (mean)	0–40	(10.6)

*Abbreviations:* TENS = Transcutaneous electrical nerve stimulation; MME = Morphine milligram equivalent.

### Safety and tolerability

In this study, all the participants who were enrolled after the positive response to diagnostic block underwent icRFA. The treatment was well tolerated, with no report of adverse effect. There was no complication after shoulder icRFA.

### Efficacy outcomes

#### Pain relief after icRFA of the SSN, AN, and LPN

**Figure 2.**
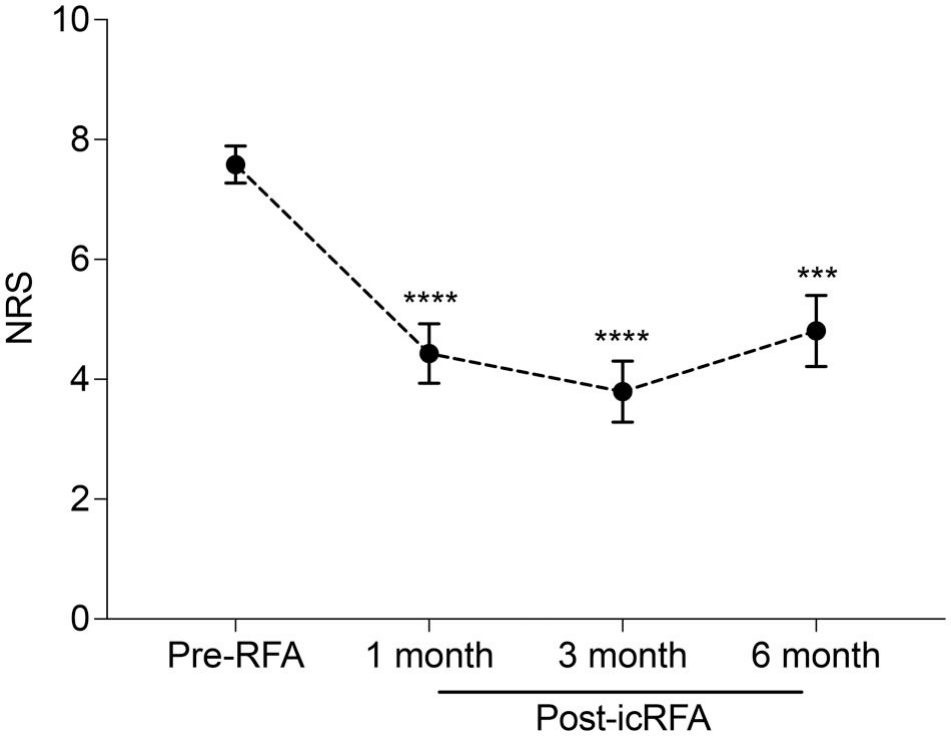
Numeric rating scale (NRS) for pain before internally cooled radiofrequency ablation (icRFA) and at 1, 3, and 6 months after icRFA. Data presented with unadjusted mean ± SEM. Mixed model for repeated measures (MMRM) analysis ***P < .0001.

Participants had an average NRS pain level of 7.58 at baseline, which significantly decreased after icRFA (*P* *<* .0001). Mean NRS pain levels were 4.43, 3.79, and 4.81 at 1 month (Adjusted [Adj.] *P* *<* .0001), 3 months (Adj. *P* *<* .0001), and 6 months (Adj. *P* = .0002) after RFA, respectively (Figure 2). The average percentage changes from baseline were 41.6%, 50%, and 36.5% at 1 month, 3 months, and 6 months after RFA, respectively, with peak pain reduction observed at the 3-month time point. The overall responder rates across any time point were 58.3% with greater than 50% maximum pain reduction and 83% with greater than 30% maximum pain reduction. The average maximum pain reduction among all participants and among responders with greater than 50% maximum pain reduction were 55.0% and 80.7%, respectively. Responder rates for greater than 50% pain reduction were 41.2%, 50%, and 30.8% at 1 month, 3 months, and 6 months after icRFA, respectively.

#### Patient-reported functional improvement after icRFA of the SSN, AN, and LPN

**Figure 3.**
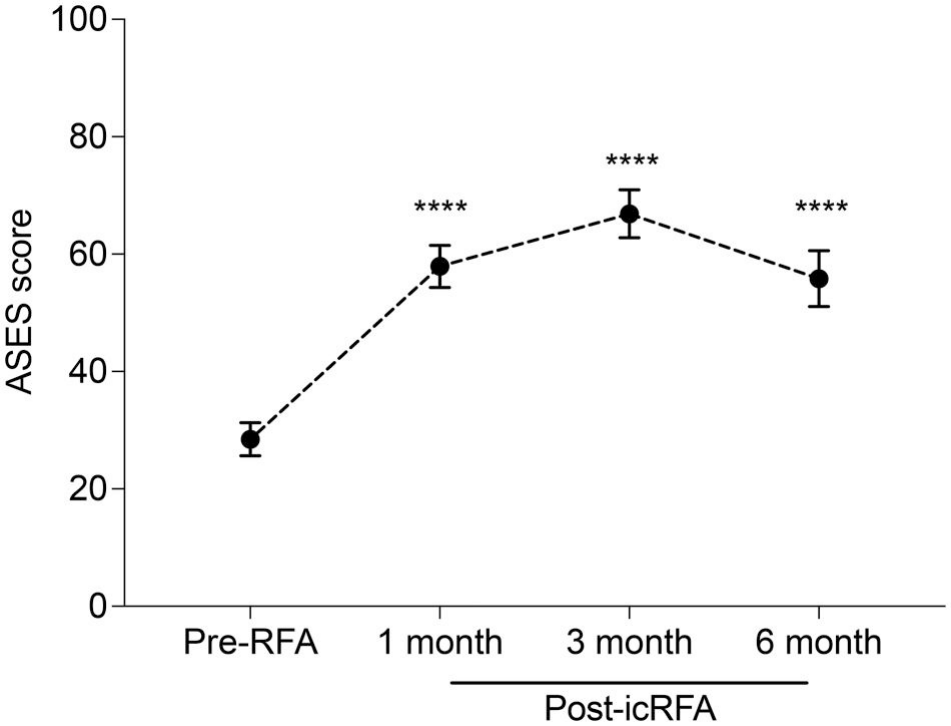
American Shoulder and Elbow Surgeons (ASES) score before internally cooled radiofrequency ablation (icRFA) and at 1, 3, and 6 months after icRFA. Data presented with unadjusted mean ± SEM. Mixed model for repeated measures (MMRM) analysis: ****P < 0.0001.

**Figure 4.**
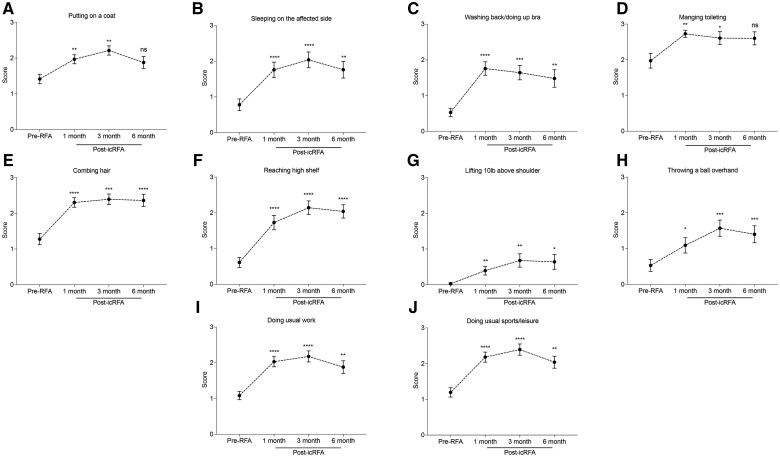
Difficulty performing activities of daily living (ADLs) before internally cooled radiofrequency ablation (icRFA) and at 1, 3, and 6 months after icRFA. Difficulty with the following was scored: **(A)** putting on a coat, **(B)** sleeping on the affected side, **(C)** washing back / doing up bra, **(D)** managing toileting, **(E)** combing hair, **(F)** reaching a high shelf, **(G)** lifting 10 lb above shoulder, **(H)** throwing a ball overhand, **(I)** doing usual work, and **(J)** doing usual sports/leisure activity; scores ranged between 0 (unable to do) and 3 (not difficult). Data presented as unadjusted mean ± SEM. Mixed model for repeated measures (MMRM) analysis: *P < .05, **P < .01, ***P < .001, ****P < .0001.

Overall ASES scores and scores in the questionnaire related to activities of daily living (ADLs) in patients with chronic shoulder pain showed statistically significant improvement after icRFA (Adj. *P* *<* .0001) (Figures 3 and 4). Specifically, the ASES score improved from 28.4 at baseline to 57.9, 66.8, and 55.8 at 1 month (Adj. *P* *<* .0001), 3 months (Adj. *P* *<* .0001), and 6 months (Adj. *P* *<* .0001) after icRFA, respectively (Figure 3). The average percentage changes in the ASES score from baseline were 104%, 135%, and 96.5% at 1 month, 3 months, and 6 months after icRFA, respectively, with the peak score observed at 3 months after icRFA. The ASES questionnaire includes 10 questions that evaluate the difficulty of performing the ADLs, with scores ranging from 3 (not difficult) to 0 (unable to perform). By 1 month after icRFA, difficulties in performing ADLs were significantly reduced across all type of ADLs (Figure 4A–H). Significant improvement in difficulties performing light activities (eg, washing back or doing bra, managing toileting, combing hair; Figure 3C–E) was achieved by 1 month after icRFA, and the improvement remained up to 6-month follow-up, whereas improvement in activities requiring large AROM or heavy-duty activities (eg, reaching a high shelf, lifting 10 lb above the shoulder, throw a ball overhand; Figure 4F–H) took 3 months to reach maximum benefit. To further evaluate the impact of icRFA on the functional outcome, we performed post-hoc analysis with 100%, 50%, and 30% improvement in ASES score as the response threshold to calculate responder rates. In this analysis, 52.9%, 64.3%, and 42.3% of patients had greater than 100% improvement in the ASES score at 1 month, 3 months, and 6 months after icRFA, respectively; 67.6%, 77.8%, and 65.4% of patients had greater than 50% improvement in the ASES score at 1 month, 3 months, and 6 months after icRFA, respectively; and 84.8%, 89.3%, and 73.1% of patients had a greater 30% improvement in the ASES score at 1 month, 3 months, and 6 months after icRFA, respectively. Of note, 91.2% of patients had greater than 30% improvement in the ASES score at some point after icRFA.

#### Objective functional improvement based on AROM after icRFA of the SSN, AN, and LPN

**Figure 5.**
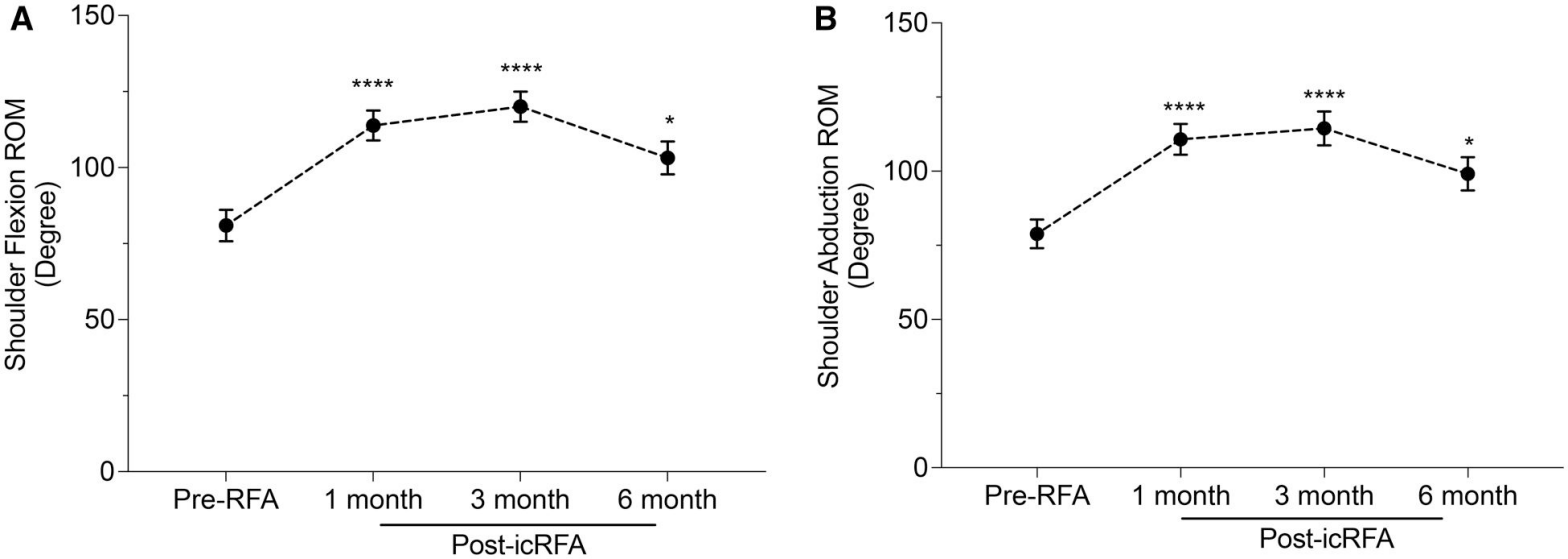
Active range of motion (AROM) before internally cooled radiofrequency ablation (icRFA) and at 1, 3, and 6 months after icRFA. Degrees of AROM for **(A)** shoulder flexion and **(B)** shoulder abduction. Data presented with unadjusted mean ± SEM. Mixed model for repeated measures (MMRM) analysis: ***P < .001, ****P < .0001.

Flexion and abduction AROM of the affected shoulder showed statistically significant improvement after icRFA (flexion *P* *<* .0001; abduction *P* *<* .0001). Flexion AROM improved from 80.9 degrees at baseline to 113.8 degrees, 120.0 degrees, and 103.2 degrees at 1 month (Adj. *P* *<* .0001), 3 months (Adj. *P* *<* .0001), and 6 months (Adj. *P* = .0139) after icRFA, respectively (Figure 5). The average percentage changes in AROM from baseline were 40.7%, 48.3%, and 27.6% at 1, 3, and 6 months after icRFA, respectively. Similarly, abduction AROM improved from 78.9 degrees at baseline to 110.8 degrees, 114.4 degrees, and 99.1 degrees at 1 month (Adj. *P* *<* .0001), 3 months (Adj. *P* *<* .0001), and 6 months (Adj. *P* *<* .0001) after icRFA, respectively. The average percentage changes in abduction AROM from baseline were 40.4%, 45.0%, and 25.6% at 1, 3, and 6 months after icRFA, respectively. The peak improvement in AROM for both flexion and abduction was observed at 3 months after icRFA. Although there was a slight reduction in AROM at 6 months compared with the peak improvement at 3 months, AROM remained significantly improved compared with pre-RFA levels. Post-hoc analysis was performed to evaluate the functional responders. In this analysis, 58.8%, 67.9%, and 42.3% of patients had greater than 30% improvement in the AROM flexion score at 1 month, 3 months, and 6 months after icRFA, respectively; 58.8%, 53.6%, and 38.5% of patients had greater than 30% improvement in the AROM abduction score at 1 month, 3 months, and 6 months after icRFA, respectively.

#### Variables associated with the therapeutic response to icRFA

**Figure 6.**
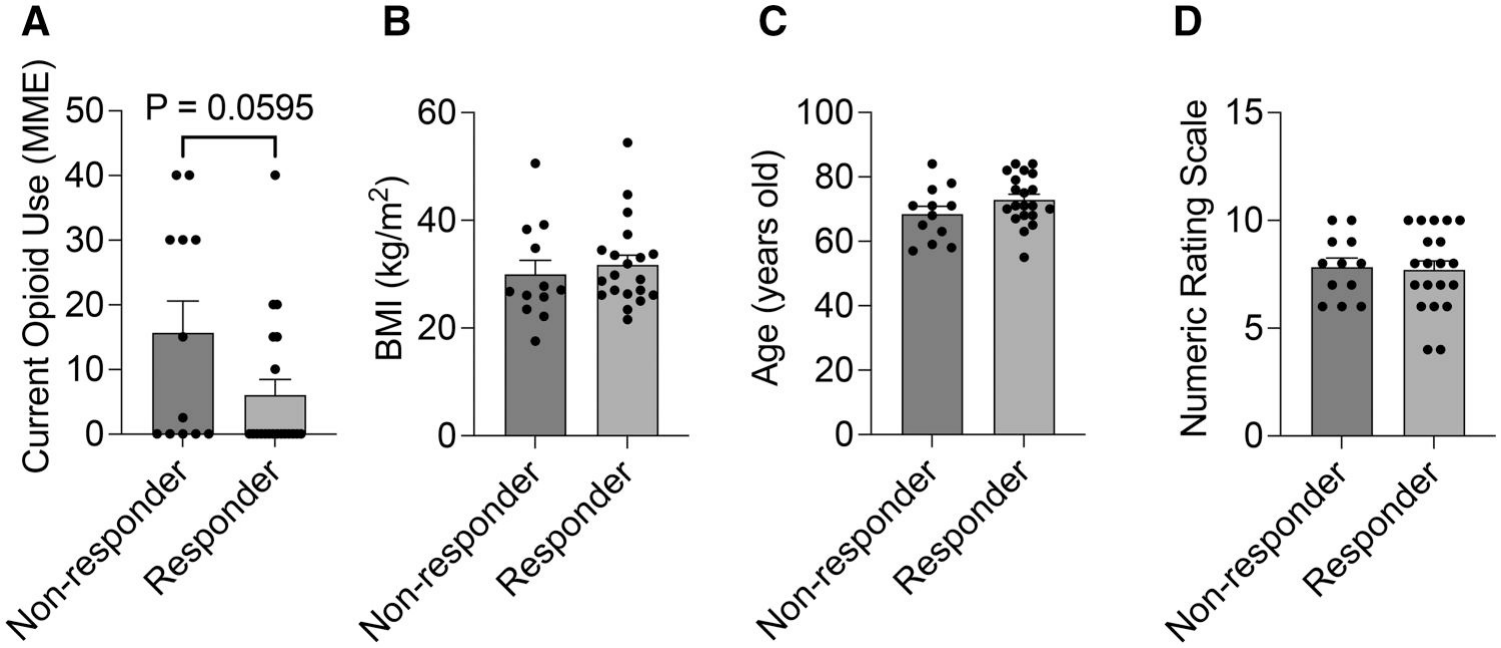
Comparison of patient characteristics between responders and nonresponders to shoulder internally cooled radiofrequency ablation (icRFA). Responders were defined as patients with ≥50% maximum pain reduction. **(A)** opioid use (morphine milligram equivalent [MME]) at the time of icRFA treatment, **(B)** body mass index, **(C)** age, and **(D)** pain level. Data presented as mean ± SEM, 2-tailed, unpaired *t* test.

**Figure 7.**
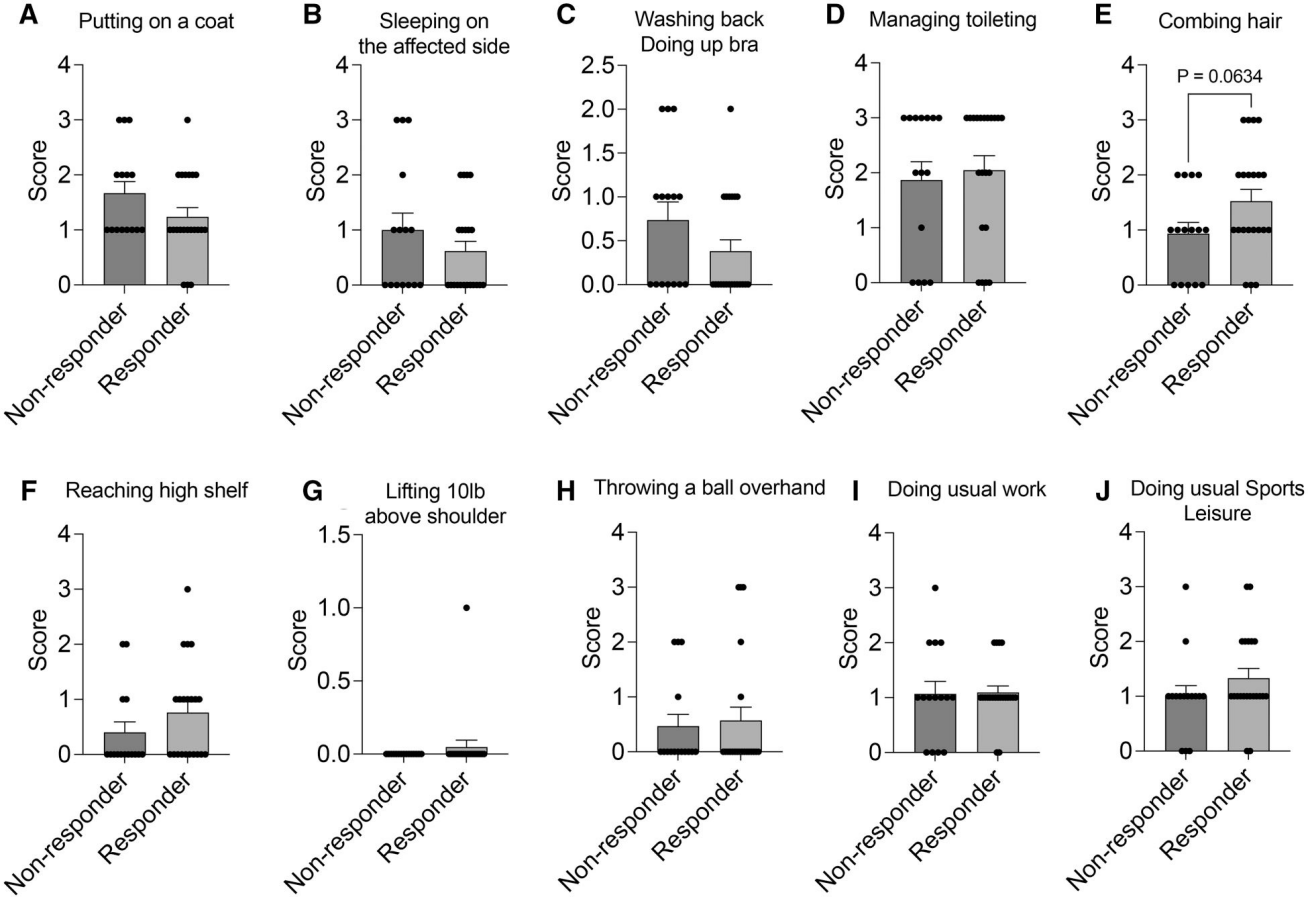
Comparison of baseline functionality between responders and nonresponders to shoulder internally cooled radiofrequency ablation (icRFA). Difficulty with the following was scored: **(A)** putting on a coat, **(B)** sleeping on the affected side, **(C)** washing back / doing up bra, **(D)** managing toileting, **(E)** combing hair, **(F)** reaching a high shelf, **(G)** lifting 10 lb above shoulder, **(H)** throwing a ball overhand, **(I)** doing usual work, and **(J)** doing usual sports/leisure activity. Scores ranged between 0 (unable to do) and 3 (not difficult). Data presented as unadjusted mean ± SEM.

Currently, there are no guidelines or studies offering insights into optimal patient selection for shoulder RFA. Although the present study was not powered for this purpose, we analyzed patient characteristics, shoulder pathology, and psychiatric conditions associated with the therapeutic response to icRFA for treating chronic shoulder pain. Continuous variable and categorical data were analyzed by unpaired *t* tests and Fisher exact tests to compare between responders (≥50% maximum pain reduction) and nonresponders to icRFA. There was no variable statistically associated with icRFA responders. None of the patient characteristics with continuous variables displayed statistically significant differences between responders and nonresponders (Figure 6). The responder rates did not differ by the presence or absence of shoulder pathologies such as glenohumeral osteoarthritis, acromioclavicular osteoarthritis, rotator cuff pathology, labral pathology, adhesive capsulitis, biceps tendinopathy, or bursitis pathology, nor did they differ by the presence or absence of psychiatric conditions in our participants (Table 3). No significant difference was observed in difficulty performing ADLs between responders and nonresponders overall (Figure 7).

**Table 3.** Comparison of patient characteristics between responders and nonresponders to shoulder icRFA: response to icRFA as related to patient characteristics and shoulder pathology.

Variable	Responders, *n*	Nonresponders, *n*	% of patients, responders	Odds ratio	95% CI	*P* value
Sex						
Male	9	3	25.71%	2.75	0.54 to 11.0	.28
Female	12	11	34.29%			
Smoking status						
Smoker	2	5	5.56%	0.21	0.038 to 1.40	.1
Nonsmoker	19	10	52.78%			
Shoulder pathology						
GH osteoarthritis	21	12	58.33%	infinity	1.31 to infinity	.06
Without GH osteoarthritis	0	3	0.00%			
AC osteoarthritis	16	11	44.44%	1.16	0.30 to 5.60	>.99
Without AC osteoarthritis	5	4	13.89%			
Rotator cuff pathology	19	12	52.78%	2.38	0.42 to 14.5	.63
Without RC pathology	2	3	5.56%			
Labral pathology	10	6	27.78%	1.36	0.34 to 4.64	.74
Without Labral pathology	11	9	30.56%			
Adhesive capsulitis	10	5	27.78%	1.82	0.44 to 7.21	.5
Without Adhesive capsulitis	11	10	30.56%			
Biceps tendinopathy	5	4	13.89%	0.86	0.18 to 3.32	>.99
Without Biceps tendinopathy	16	11	44.44%			
Bursitis pathology	7	5	19.44%	1	0.28 to 4.12	>.99
Without Bursitis pathology	14	10	38.89%			
Psychiatric condition						
Anxiety disorder	3	2	8.33%	1.08	0.20 to 6.76	>.99
Without anxiety disorder	18	13	50.00%			
Mood disorder	3	4	8.33%	0.46	0.10 to 2.01	.42
Without mood disorder	18	11	50.00%			

*Abbreviations:* AC = acromioclavicular; CI = confidence interval; GH = glenohumeral; RC = rotator cuff.

#### Relationship between pain reduction and functional improvement

**Figure 8.**
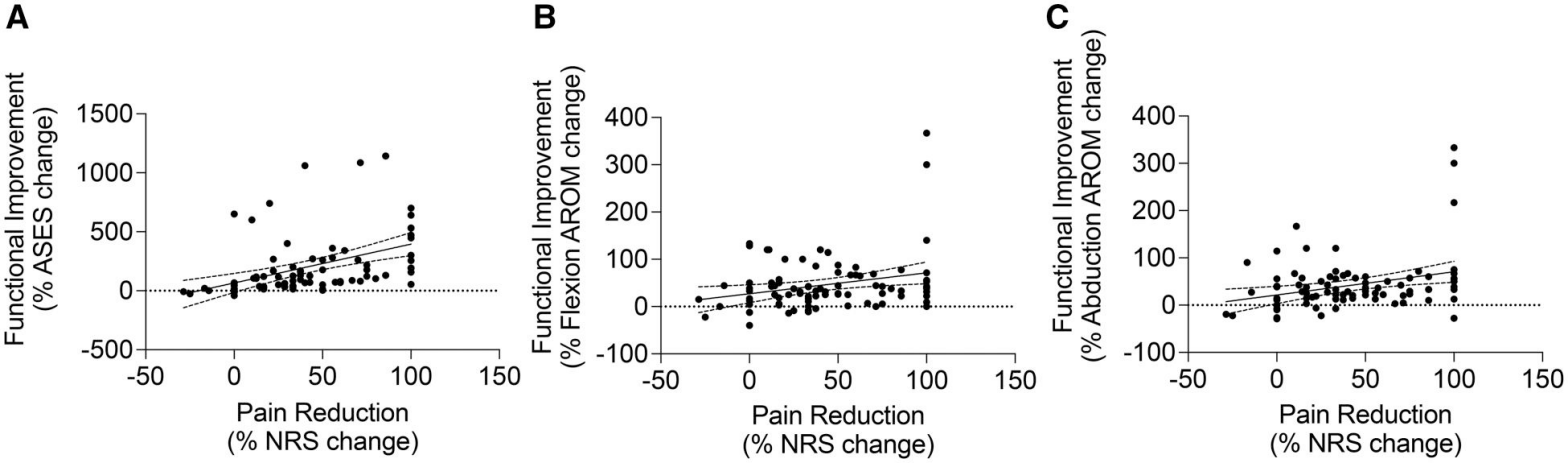
Correlation between improvement in pain levels and shoulder function.

To analyze how pain reduction correlates with functional improvement, we plotted the percentage changes in NRS against the percentage changes in ASES score, flexion AROM, and abduction AROM. We performed Spearman correlation analysis to assess the relationship between pain reduction and the functional changes. All metrics of functional improvement (ASES, flexion AROM, abduction AROM) displayed significant correlation with pain reduction (ASES *R* = 0.609, *P* *<* .0001; flexion AROM *R* = 0.228, *P* = .032; abduction AROM *R* = 0.248, *P* = .0204) (Figure 8).

## Discussion

Since its initial application in 1931 for the treatment of trigeminal neuralgia, RFA has been applied to various pain conditions, including those affecting the spine, sacroiliac joint, hip, knee, and shoulder. The potential advantage of RFA includes its longer-lasting effects, minimal side effects, and reduced tissue destruction compared with other treatments. The most comprehensive evidence to date comes from a 2021 systemic review and meta-analysis by Pushparaj et al., who analyzed 42 studies. Of these, 36 used pRFA, with 29 studies targeting only the SSN.^20^ pRFA has gained favor because of its nondestructive, neuromodulatory mechanism, which avoids the risk of compromising motor function.^30^^,^^31^ However, there is currently limited literature on the use of icRFA for chronic shoulder pain. Notably, our study found no reports of weakness or other neurological deficits after icRFA.

Previous studies have primarily targeted the SSN, which is believed to provide approximately 70% of shoulder innervation, with the remaining innervation arising from other sensory branches, such as the lower subscapular nerve, AN, and LPN.^32–36^ A previous publication by Tran et al. evaluated 12 patients with shoulder osteoarthritis who underwent icRFA targeting these SSN, AN, and LPN after successful diagnostic blocks.^37^ Similar to our study, Tran et al. used the ASES score and the visual analog scale (VAS) to assess treatment efficacy. Their findings demonstrated significant improvement in the ASES score (282% improvement in ASES score and 75% reduction in VAS at 6 months). The results are comparable to our study, although the magnitude of improvement in ASES score (282% vs 135%) and pain reduction (75% vs 50%) were greater in the study by Tran et al. Similarly, Santi et al. also demonstrated significant pain relief after icRFA of the SSN, AN, and LPN, at a level comparable to ours (50% reduction on the NRS). Our procedural approach is very similar, if not identical, to these studies. Therefore, the difference in the outcome might be attributable to variations in baseline patient characteristics, such as starting pain levels, disability, and severity of the shoulder pathology. Moreover, our study included a broader range of shoulder pathologies beyond glenohumeral osteoarthritis, making our study more generalizable to the diverse patient population commonly seen in pain clinics.

Determining the superiority of the ablating the SSN, AN, and LPN compared with the SSN alone requires further head-to-head study in the future. A recently published protocol includes the upper subscapular nerve in addition to the SSN, AN, and LPN, adding more variation to this approach.^38^ Future research should assess the onset, degree, and duration of improvement to evaluate whether targeting multiple nerves offers superior benefit. Additionally, given the heterogeneity of shoulder pathology among patients with chronic shoulder pain, it is essential to investigate whether certain patient groups benefit more from multiple-nerve ablation over single-nerve ablation.

Our study demonstrates the efficacy and safety of icRFA targeting the shoulder joint nerves, with improvements in pain scores, shoulder function, and AROM lasting for at least 6 months. Pain relief and functional improvement were observed as early as 1 month after treatment, reaching peak efficacy at 3 months, which provides a general timeline for patient expectations. icRFA can be used in conjunction with steroids to reduce steroid exposure, avoid shoulder surgery, and manage patient symptoms. Although we did not identify statistically significant factors distinguishing responders from nonresponders, trends suggest that lower opioid use, the presence of glenohumeral osteoarthritis, and less difficulty with light activities such as combing hair might be potential predictors of a positive response to icRFA; this warrants further investigation. Increasing the diagnostic threshold to 80% might improve the responder rate for pain relief but also potentially exclude patients who could benefit functionally. Appropriately determining the threshold for diagnostic response to consider moving forward with icRFA requires additional study, assessing its false-positive and false-negative rates. Avoiding inadvertent intra-articular injection of diagnostic block is likely important, as it has been shown to have less prognostic value in other diagnostic blocks, such as medial branch block for axial back pain.^39^

As expected, our correlation analysis revealed a significant relationship between pain improvement and better functional outcome, particularly with ASES score. However, we also observed cases in which functional improvement was disproportionate to the pain relief or occurred without significant pain reduction. This observation suggests that evaluating pain level alone might not fully capture the benefit of icRFA on a patient’s quality of life. Consistent with this notion, a greater number of patients experienced functional improvement compared with pain reduction across all follow-up time points. Therefore, pain relief and functional improvement should be separately considered as indications for repeat icRFA treatment in the future.

This study has several limitations that should be considered. The lack of control group, given the prospective observational nature of this study, limits our ability to compare the efficacy of icRFA with that of other conservative treatments, such as steroid injection or medical management alone. Additionally, the ASES questionnaire is subjective to variability because of subjective interpretation by the participants and framing by the evaluators. Nevertheless, the inclusion of AROM as an objective, quantifiable measure of shoulder function strengthens our confidence in the benefit of icRFA. Further research powered adequately with larger sample sizes is needed to identify factors predictive of the therapeutic response.

Despite these limitations, our study is significant, as it provides both subjective and objective evidence supporting the benefit of shoulder icRFA. The findings offer offers strong support for the efficacy of icRFA in alleviating pain and improving shoulder function across a wide range of shoulder pathologies.

## Conclusion

In summary, this study presents evidence supporting icRFA as a safe and effective therapy for chronic shoulder pain, offering mid-term improvement in patients’ symptoms and ADLs, both qualitatively and quantitatively. Our findings advocate for the use of icRFA in treating chronic shoulder pain that is refractory to physical therapy, pharmacotherapy, and intra-articular steroid injection.

## Data Availability

All the datasets used and analyzed in this study are available from the corresponding author upon request.
